# Up-Regulation of Activating Transcription Factor 3 in Human Fibroblasts Inhibits Melanoma Cell Growth and Migration Through a Paracrine Pathway

**DOI:** 10.3389/fonc.2020.00624

**Published:** 2020-04-21

**Authors:** Tingjian Zu, Jie Wen, Lin Xu, Hui Li, Jun Mi, Hui Li, Cord Brakebusch, David E. Fisher, Xunwei Wu

**Affiliations:** ^1^Department of Tissue Engineering and Regeneration, School and Hospital of Stomatology, Shandong University, Jinan, China; ^2^Shandong Key Laboratory of Oral Tissue Regeneration and Shandong Engineering Laboratory for Dental Materials and Oral Tissue Regeneration, Jinan, China; ^3^Department of Orthodontics, Liaocheng People's Hospital, Liaocheng, China; ^4^Department of Dermatology, Qilu Hospital of Shandong University, Jinan, China; ^5^Department of Hematology, Southwest Hospital, Third Military Medical University, Chongqing, China; ^6^Biotech Research and Innovation Centre (BRIC), University of Copenhagen, Copenhagen, Denmark; ^7^Cutaneous Biology Research Center, Massachusetts General Hospital and Harvard Medical School, Boston, MA, United States

**Keywords:** melanoma, fibroblasts, stromal cells, activating transcription factor 3, interleukin 6

## Abstract

The treatment of melanoma has remained a difficult challenge. Targeting the tumor stroma has recently attracted attention for developing novel strategies for melanoma therapy. Activating transcription factor 3 (ATF3) plays a crucial role in regulating tumorigenesis and development, but whether the expression of ATF3 in human dermal fibroblasts (HDFs) can affect melanoma development hasn't been studied. Our results show that ATF3 expression is downregulated in stromal cells of human melanoma. HDFs expressing high levels of ATF3 suppressed the growth and migration of melanoma cells in association with downregulation of different cytokines including IL-6 *in vitro*. *In vivo*, HDFs with high ATF3 expression reduced tumor formation. Adding recombinant IL-6 to melanoma cells reversed those *in vitro* and *in vivo* effects, suggesting that ATF3 expression by HDFs regulates melanoma progression through the IL-6/STAT3 pathway. More importantly, HDFs pretreated with cyclosporine A or phenformin to induce ATF3 expression inhibited melanoma cell growth *in vitro* and *in vivo*. In summary, our study reveals that ATF3 suppresses human melanoma growth and that inducing the expression of ATF3 in HDFs can inhibit melanoma growth, a new potential melanoma therapeutic approach.

## Introduction

Human cutaneous melanoma originates from dysregulated proliferation of melanocytes and is one of the most aggressive and deadly forms of skin cancer, accounting for more than 80% of skin cancer-related deaths (1). Despite recent advances in melanoma therapy including the use of immune checkpoint inhibitors (2, 3), treatment options for metastasized melanoma are still very insufficient (4). The activating BRAF mutation has been considered to be a main factor driving the initiation of melanoma (5). However, ~80% of benign melanocytic nevi contain BRAF-activating mutation, although only a small percentage of them will develop into malignant melanoma (6). The mechanism underlying malignant transformation from nevus to melanoma is therefore still unclear.

Recent research studies have shown the changes of stroma cells play essential roles in tumor initiation (7, 8). The main component of the tumor microenvironment is the activated fibroblasts in cancers, which called cancer-associated fibroblasts (CAFs) (9). A large number of studies demonstrated that CAFs secrete numerous cytokines and growth factors which promote tumor growth and metastasis (10–13). Kim's (14) research showed that senescent CAFs can promote melanoma initiation and development, suggesting that tumor microenvironment changes may play important roles in melanoma initiation and development. Therefore, targeting CAF may provide a potential new therapeutic approach for treatment of melanoma in the clinic (3, 15).

Activating transcription factor 3 (ATF3) is a key transcription factor in cellular stress responses, with different expression levels and functions in different tissues (16). It has been shown that ATF3 can be either an oncogene or a tumor suppressor gene depending on the type of tumor (17–21). For instance, ATF3 was found to be down-regulated in esophageal squamous cell carcinoma (ESCC) lesions, and forced expression of ATF3 led to decreased growth and invasive properties of ESCC cells *in vitro* and *in vivo* through regulation of MDM2 expression (22). On the other hand, we described earlier that upregulation of ATF3 in human skin epidermal cells blocks p53-dependent senescence to promote tumorigenesis in the skin (23). ATF3 is also expressed in human dermis, but hardly anything is known about its function in human dermal fibroblasts (HDFs) or melanoma-associated fibroblasts. Therefore, the present study aims to investigate whether the expression level of ATF3 in dermal fibroblasts can affect melanoma cell growth and migration.

## Materials and Methods

### *In vitro* Cell Culture

Primary HDFs were isolated from foreskin tissues following a protocol described previously (24, 25). The HDFs and human melanoma cell lines Mel-JuSo and UACC62 were cultured in Dulbecco's Modified Eagle's medium (DMEM, Gibco, USA) supplemented with 10% FBS (Biological Industries, Israel), penicillin (100 units/ml) and streptomycin (100 μg/ml) (ThermoFisher, USA) (complete medium) at 37°C in a humidified 5% CO_2_ atmosphere.

### RNA Extraction and Real-Time Quantitative Reverse Transcription PCR (qRT-PCR)

Total RNA was extracted from HDFs and melanoma cells using a Takara MiniBEST Universal RNA Extraction Kit (Takara, Japan), and 1 μg of total RNA was reverse transcribed into cDNA using a Primer Script RT Reagent Kit (Takara, Japan). All qRT-PCR amplification cycles were performed using SYBR Premix Ex Taq (Tli RNaseH Plus) (Takara, Japan) with a Light Cycler 480 II (Roche Diagnostics, Mannheim, Germany) according to the manufacturer's protocol. Amplification conditions were set to an initial step of 95°C for 30 s followed by 45 cycles of 95°C for 5 s, 60°C for 35 s, 72°C for 60 s, and then a final step of 40°C for 30 s. All RNA samples were analyzed in triplicate with gene-specific primers along with primers for human ribosomal protein mRNA *RPLP0* (H36B4), which was used as a housekeeping gene for normalization. The list of gene-specific primers is provided in Supplementary Table 1.

### Western-Blot Analysis

Cells were harvested at the indicated time points and rinsed with ice-cold PBS. Cells were lysed in radioimmunoprecipitation assay buffer (RIPA buffer, Beyotime Institute of Biotechnology, Shanghai, China) containing 1% phenylmethylsulfonyl fluoride (PMSF, Beyotime Institute of Biotechnology, Shanghai, China) for 30 min on ice and then centrifuged at 12,000 rpm for 15 min at 4°C. The protein concentration of each sample was measured using a bicinchoninic acid assay kit (BCA Protein Assay Kit, Beijing Solarbio Science & Technology). Equal amounts (30 μg) of protein samples were electrophoresed in 10 or 12% sodium dodecyl sulfate-polyacrylamide gels (Beyotime Institute of Biotechnology) and electrically transferred to 0.45 μm polyvinylidene fluoride (PVDF) membranes (Merck Millipore, USA). The membranes were blocked with 5% non-fat milk at room temperature for 1 h and then incubated with primary antibodies at 4°C overnight. GAPDH was used as loading control. The next day, the membranes were washed with Tris-based saline-Tween-20 (TBS-T, 20 mmol/ml Tris-HCl, 150 mmol/ml NaCl and 0.05% Tween 20) three times for 10 min each, and then the membranes were incubated with secondary antibodies for 1 h at room temperature. The protein bands were visualized using a Chemiluminescent HRP Substrate Kit (Millipore, Billerica, MA, USA) and MiniChem 610 Image system (Sagecreation, Beijing). The levels of protein expression were normalized to the corresponding GAPDH bands using ImageJ software. The list of primary and secondary antibodies is provided in Supplementary Table 2.

### Generation of Conditioned Media

To prepare conditioned media (CM), HDFs with or without ATF3 overexpression were cultured to reach 80% confluence in 10 cm dishes, then the medium was replaced with fresh complete medium. Alternatively, either phenformin (1.5 mM) or cyclosporine A (10 μM) was added to an HDF culture for 24 h before replacing the medium with fresh drug-free medium. Forty-eight hours later, the media were collected and filtered through 0.22 μm PES filter (Merck Millipore Ltd. USA) to remove cells and cellular debris. The media were used immediately or aliquoted and kept at −80°C for later use for culture of melanoma cells.

### ELISA

ELISA assays were carried out using R&D Systems kits according to the manufacturer's protocols. Briefly, total protein concentrations in CM were measured using a BCA Protein Assay Kit (Solarbio Science & Technology, Beijing), then CM were diluted to suitable concentrations for measurements of IL-6, IL-8, and TNFα using the following ELISA kits: Human IL-6 ELISA Kit (Cat. No. VAL102, R&D Systems, USA), human IL-8 ELISA Kit (Cat. No. VAL103, R&D Systems, USA) and human TNFα ELISA Kit (Cat. No. VAL105, R&D Systems, USA). Absorbance at 450 nm was measured using a plate reader (Spectrostar Nano, BMG Labtech) and the concentrations of IL-6, IL-8, and TNFα in CM were calculated from the respective standard curves.

### Immunofluorescence and Immunohistochemical Staining

Immunofluorescence (IF) and immunohistochemical (IHC) staining were performed followed standard protocols. Five micrometer thick sections were cut from formalin-fixed paraffin-embedded clinical tissue blocks and the sections on slides were deparaffinized, rehydrated, immersed in 0.3% hydrogen peroxide to block endogenous peroxidase, and then submerged in EDTA antigen retrieval solution (ZSGB-BIO, Beijing) for heat-induced antigen retrieval according to the manufacturer's protocol. After blocking with 10% goat serum, the slides were incubated with primary antibodies at 4°C overnight. On the second day, the slides were washed with PBS (3 × 10 min). For IF staining, the slides were incubated for 1 h with DyLight488 goat anti-mouse IgG(H+L) (Multi Science, Hangzhou) and DyLight594 goat anti-rabbit IgG(H+L) (Multi Science, Hangzhou) at room temperature in the dark. Sections were then incubated with 4', 6-diamidino-2-phenylindole (DAPI, Abcam, USA) to stain nuclei. For IHC staining, the slides were incubated for 1 h with rabbit biotin-streptavidin HRP secondary antibody (ZSGB-BIO, Beijing). After washing 3 times with PBS, the sections were incubated with DAB staining solution (ZSGB-BIO, Beijing) for 5 min, counterstained with hematoxylin, dehydrated and mounted. An immunofluorescence microscope (Olympus BX53-DP80, Tokyo, Japan) was used for analysis of staining.

For quantification of ATF3 nuclear staining, has been linked to its activation (26–28), all staining of clinical tumor tissues was performed twice and was evaluated by two independent investigators. Evaluations were based on arbitrary units as follows: 0: no staining; 1: intermediate (weak and incomplete) nuclear staining; 2: strong, complete nuclear staining. For the relative quantification of ATF3 expression, the mean value of the independent measurements was taken as the final score.

### RNAscope *in situ* Hybridization Analysis

*In situ* hybridization with ATF3-specific RNAscope Probe-Hs-ATF3 (Cat. No.470861, Advanced Cell Diagnostics, Inc. USA) was performed according to the RNAscope 2.5 RED Assay Protocol (#322452 and #322360). Briefly, paraffinized tissue slides were baked at 60°C for 1 h and then deparaffinized. Sections were immersed in RNAscope Hydrogen Peroxide for 10 min and then submerged in RNAscope Target Retrieval Reagent at 100°C for 15 min for target retrieval. Sections were then incubated in RNAscope Protease Plus reagent in a HybEZ II hybridization oven at 40°C for 30 min. Tissue slides were then incubated with RNAscope Probe-Hs-ATF3, or with RNAscope Negative Control Probe dapB (Cat. No. 310043) as negative control or RNAscope Probe Hs-PPIB (Human-Peptidylprolyl Isomerase B) (Cat. No. 313902) as positive control (as shown in Supplementary Figure 1), in the hybridization oven at 40°C for 2 h, followed by successive incubations with Hybridize Amp 1–6 reagents. Then the signals were detected with RNAscope 2.5 Red signal detecting reagent and the tissues were counterstained with hematoxylin solution and mounted. Tissues exhibiting red-dyed points were considered positive.

### Cell Proliferation Assay

Melanoma cells were seeded in 96-well plates at a density of 2,000 cells/well in triplicate and grown overnight in complete medium. Then the medium in all wells was replaced with CM every 48 h. At every assay time point, 10 μl of Cell Counting Kit-8 (CCK-8) (Dojindo, Japan) working solution was added to each well and cultured for 1.5 h at 37°C. Absorbance at 450 nm was measured using a plate reader (Spectrostar Nano, BMG Labtech). The experiments were repeated at least three times.

### Colony Formation Assay

Melanoma cells were plated in 6-well plates at 1,500 cells/well and grown overnight. The cell culture medium was replaced with CM every 2 days for 14 days. At the end of the cell culture, cells were washed with PBS, fixed with 4% paraformaldehyde solution (Sigma-Aldrich, USA) and stained with 0.1% crystal violet (Solarbio Science & Technology, Beijing). Cell colonies with more than 50 cells were counted under an inverted microscope (Olympus CKX41, Tokyo, Japan). The experiments were performed in triplicate and repeated at least three times.

### Co-culture Assay

The co-culture assay was performed using cell culture inserts with 3 μm pore diameter membranes (Millipore, Cat. No. MCSP12H48, USA) in 12-well tissue culture plates. AT3-overexpressing or control HDFs (100,000 cells in 1 ml DMEM medium containing 3% FBS) were seeded on the bottom wells of the plate and 20,000 melanoma cells in 300 μl DMEM medium containing 0.5% FBS were seeded in the cell culture inserts in the wells. At every assay time point, melanoma cells on insert membranes were collected by trypsin digestion and counted with both a Countstar IC 1000 automated cell counter (ALIT Life Science Co. Limited) and a hemocytometer. The experiments were performed in triplicate and repeated at least three times.

### Cell Migration Assay

The cell migration assay was performed using cell culture inserts with 8 μm pore diameter membranes (Falcon, USA) in 24-well plates. 60,000 ATF3-overexpressing or control HDFs in 500 μl of complete medium were seeded per well and cultured for 48 h, or 500 μl per well of conditioned medium was added to empty wells just before adding melanoma cells to the cell culture inserts. 40,000 melanoma cells in 200 μl DMEM medium containing 0.5% FBS were seeded in the inserts placed in the wells of the 24-well plates. The plates were incubated at 37°C for 24 h. Cells that migrated through the insert membrane were fixed with 4% paraformaldehyde solution (Sigma-Aldrich, USA) and stained with 0.1% crystal violet (Solarbio Science & Technology, Beijing). Images of five randomly selected fields of the fixed cells were captured and the cells were counted. The experiments were repeated at least three times.

### Wound Healing Assay

Melanoma cells were incubated in complete medium in 6-well plates. When the cells reached more than 90% confluency, the cell monolayer was scratched with a sterile 10 μl pipette tip (time 0), washed with PBS to remove the cells within the linear scratch and refreshed with CM. Pictures of 5 non-overlapping fields were photographed at 0 h and 24 h using an inverted microscope (Olympus CKX41, Tokyo, Japan). The percentage of wound healing was reported as the healing area width divided by the total wound area width. The experiments were repeated at least three times.

### Construction of ATF3-Targeting CRISPR/Cas 9

The oligonucleotide sequences of two ATF3 small guide RNAs (sgRNA1, sgRNA2) were designed and identified using the Broad Institute CRISPR design tool (http://crispr.mit.edu/). All 20-bp sequences preceding an NGG protospacer-adjacent motif were analyzed for potential off-target sites in the human genome using BLAST searches of whole genome DNA sequences. We purchased the lentiviral vector pSpCas9(BB)-2A-Puro (PX459) V2.0 (Plasmid #62988), which expresses SpCas9 under the transcriptional control of a cytomegalovirus promoter, from Addgene and sgRNA1 and sgRNA2 were cloned into the plasmid according to the protocol published by Feng Zhang's Lab (29). The upstream and downstream oligonucleotides for each sgRNA were phosphorylated, annealed, and then subcloned into the pSpCas9 (BB)-2A-Puro (PX459) V2.0 vector. The ATF3 sgRNA1 and sgRNA2 sequences used in this study are provided in Supplementary Table 1.

### Virus Preparation and Infection

Retrovirus and lentivirus preparation and infection were performed as described previously (23). HEK 293T cells were transfected with viral expression vector together with packaging vector using Lipofectamine 3000 Kit (Invitrogen, USA) following the manufacturer's protocol, and virus particles were collected at 24, 48, and 72 h after transfection. HDFs were seeded in 10 cm dishes and incubated until the cell density reached 60–80%, and then the cells were incubated with 6 ml virus plus 5 μl polybrene solution (10 mg/ml, Sigma-Aldrich, USA) for 6 h. The virus-containing medium was then removed, the cells were washed with PBS, and then the HDFs were incubated with normal DMEM growth medium supplemented with 1 μg/ml puromycin (Sigma-Aldrich, USA) for 48 h to select for transduced cells. The surviving fibroblasts were validated for ATF3 deletion by Western blot.

### *In vivo* Tumor Assay

Tumor xenografts were grown and measured as described previously (23). To test the inhibitory effect of ATF3-overexpressing HDFs plus/minus IL-6 treatment on melanoma cell growth *in vivo*, 8 week-old female nude/nude mice (Charles River Laboratory, Wilmington, MA, USA) were randomly assigned to three groups: (1) the control NEO group (NEO) with subcutaneous injection of UACC62 melanoma cells mixed with NEO-expressing HDFs plus intraperitoneal injection of PBS after grafting, (2) the AFT3 overexpression group (ATF3-OE) with injection of UACC62 cells mixed with ATF3-overexpressing HDFs plus intraperitoneal injection of PBS after grafting, and (3) the rhIL-6 treatment group with injection of UACC62 cells mixed with ATF3-overexpressing HDFs plus intraperitoneal injection of recombinant human IL-6 (Cat. No. Z03034-10, GenScript, Nanjing) at 100 ng/kg/day/mouse (3 mice per group). Each injection contained 0.5 million UACC62 melanoma cells and 0.5 million HDFs, and each mouse received injections at 4 different sites. Three weeks after grafting, mice were euthanized for measurements of tumor volumes and tumor weights.

To test whether HDFs pretreated with cyclosporine A (CsA) affect melanoma cell growth, HDFs were treated with either 10 μM CsA or DMSO (as control) for 24 h, then the cells were collected and mixed with UACC62 melanoma cells at a 1:1 ratio. The cell mixture was injected subcutaneously into the back skin of 8-week-old female nude/nude mice at 4 different sites (total of 1 million cells per injection site). Four mice per group were used and the mice were euthanized 3-weeks after grafting for analysis of tumor volumes and tumor weights. The *in vivo* experiments were performed 3 times.

### Statistical Analysis

All quantification data are summarized as the mean ± standard error of the mean (SEM) of at least three biological replicates of each experiment in all cases. GraphPad Prism 6 (GraphPad Software Inc., LaJolla, CA, USA) was used for statistical analysis. Student's *t*-test was used for comparison of 2 groups; one-way or two-way ANOVA with correction for multiple pairwise comparisons was used when comparing more than 2 groups with one or two independent variables, respectively. The *p*-values from the statistical analyses are indicated in the figures.

## Results

### Clinical Melanoma Stromal Cells Express Low Levels of ATF3

**Figure 1:**
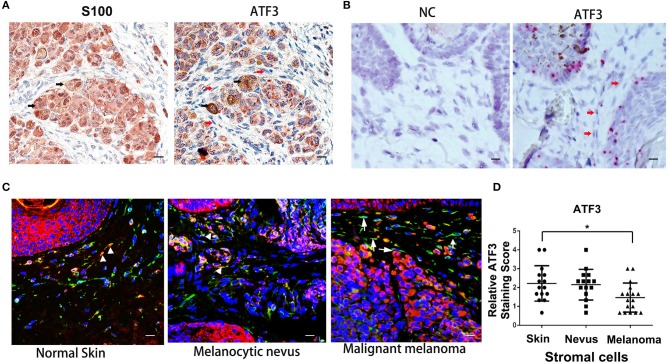
Low expression of ATF3 in human melanoma stromal cells. **(A)** Immunohistochemistry staining for S100 and ATF3 in clinical melanoma tissues. Black arrows indicate melanoma cells and red arrows indicate stromal cells. **(B)** RNAscope *in situ* hybridization staining for *ATF3* and negative control (NC) in melanoma samples. Red arrows indicate stromal cells. **(C)** Double immunofluorescence staining of ATF3 (red) and vimentin (green) in normal skin, melanocytic nevus and melanoma tissues. White arrowheads indicate stromal cells with positive staining of both vimentin (green) and ATF3 (red); white arrows indicate stromal cells with vimentin positive and ATF3 negative staining. **(D)** Relative scores of ATF3 protein expression levels in stromal cells, detected by vimentin staining as in **(C)** and quantified nuclear staining of ATF3 from all staining of 15 normal human skin, 15 melanocytic nevus and 17 melanoma tissues. Data are presented as the mean ± standard deviation, *p < 0.05. All scale bars represent 20 μm.

To investigate the expression level of ATF3 in human melanoma stromal cells, clinical melanoma samples were analyzed by immunohistochemistry staining with S100 (30–32) and ATF3 antibodies. Tumor cells identified by positive S100 staining (black arrows, left panel, Figure 1A) expressed clearly detectable amounts of ATF3 (black arrows, right panel, Figure 1A), while melanoma stromal cells identified by their spindle shapes and absence of S100 protein displayed extremely weak ATF3 staining (red arrows, right panel, Figure 1A). Next, RNAscope technology was performed to detect *ATF3* mRNA within intact cells. We found strong *ATF3* signals in the tumor cells but not in the stromal cells, as indicated in Figure 1B (right panel, red arrows), and no positive staining with the negative control probe (Figure 1B, left panel). Finally, double immunofluorescence staining of vimentin and ATF3 was carried out in melanoma tumor samples, melanocytic nevi and normal skin tissues. As shown in Figure 1C, we observed in benign nevi and normal skin tissues that vimentin-expressing stromal cells expressed ATF3 (stained red, white arrow heads, Figure 1C), while the majority of vimentin+ cells in malignant melanoma did not express detectable ATF3 (white arrows, Figure 1C). Quantitative analysis of ATF3 nuclear staining indicated with DAPI (blue) in stroma cells, which recognized by vimentin positive staining confirmed that the lowest ATF3 staining was in the stroma cells of the malignant melanoma tumor tissues (Figure 1D). These data suggest that the decreased expression of ATF3 in the tumor stromal cells correlate with the development of malignant melanoma.

### HDFs Overexpressing ATF3 Inhibit Melanoma Cell Growth and Migration in Co-culture Assays

**Figure 2:**
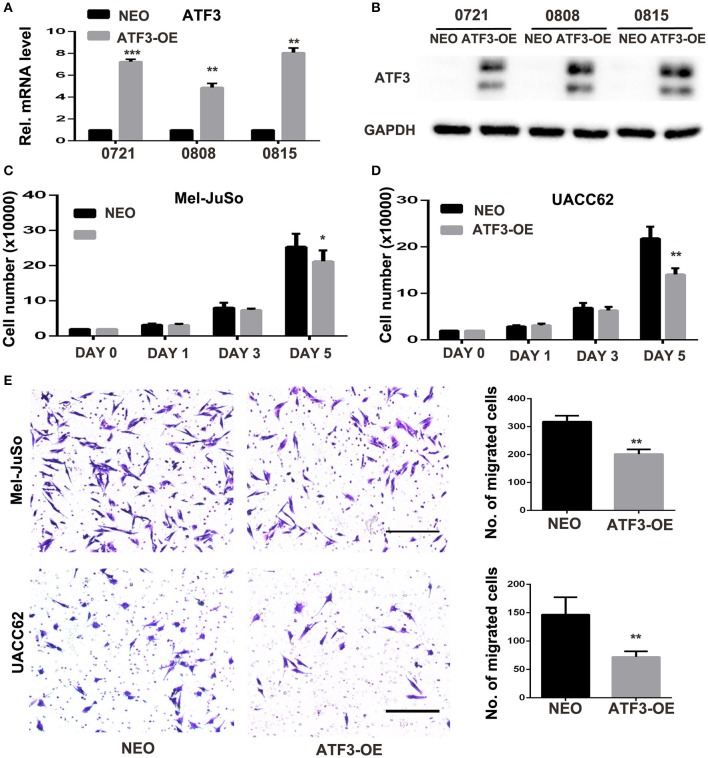
ATF3-overexpressing human dermal fibroblasts (HDFs) inhibited melanoma cell proliferation and migration in co-culture assays. **(A)** qRT-PCR analysis of *ATF3* mRNA levels and **(B)** Western blot analysis of ATF3 protein with GAPDH as loading control, in three HDF strains infected with ATF3-expressing (ATF3-OE) or control neomycin (NEO)-expressing retrovirus. **(C,D)** The numbers of Mel-JuSo **(C)** and UACC62 **(D)** melanoma cells after co-culture for the indicated number of days with the 3 HDF strains overexpressing ATF3 (ATF3-OE) or expressing NEO. **(E)** Images (left panels) and quantification (graphs) of Mel-JuSo and UACC62 cells that had migrated after co-culture for 24 h with ATF3-OE or control NEO HDFs. Bars represent 100 μm. Data are presented as the mean ± standard deviation, *p < 0.05, **p < 0.01, ***p < 0.001.

Next, we investigated the effects of ATF3 overexpression in fibroblasts on melanoma function *in vitro*. ATF3 was stably overexpressed (ATF3-OE) in three different primary HDF cultures by retroviral transduction, resulting in 5–8-fold increased expression of ATF3 in the three strains compared with mock (NEO) transduced cells (Figures 2A,B). In order to minimize artificial effects induced by overexpressed ATF3, the HDF line with the lowest expression of ATF3 after virus infection (strain 0808) was used for the following studies. A co-culture assay shows that the numbers of Mel-JuSo and UACC62 melanoma cells co-cultured with ATF3-overexpressing HDFs were significantly less than the numbers of melanoma cells co-cultured with control HDFs (Figures 2C,D). Then, we tested the effects of ATF3 overexpression in HDFs on melanoma cell migration. As shown in Figure 2E, both Mel-JuSo and UACC62 melanoma cells migrated significantly less when co-cultured with ATF3-overexpressing HDFs than when co-cultured with NEO-expressing HDFs. These results suggest that increased expression of ATF3 in HDFs can inhibit both growth and migration of melanoma cells.

To test the effect of reducing ATF3 expression in HDFs, we engineered deletion of the *ATF3* gene by lentiviral-mediated CRISPR/Cas9 genome editing. Efficient loss of ATF3 protein was confirmed by Western blot (Supplementary Figure 2A). Co-culture of *ATF3*-deleted HDFs with Mel-JuSo or UACC62 melanoma cells did not affect melanoma cell growth or migration (Supplementary Figures 2B–D), suggesting that endogenous ATF3 levels in *in vitro* cultured HDFs are insufficient to affect melanoma growth or migration significantly. This result agrees with a recent *in vivo* study by Avraham *et al*. showing that the growth of Lewis Lung Carcinoma cells was not affected when transplanted onto *ATF3* knockout mice, but was promoted when transplanted onto *ATF3* and *JDP2* (c-Jun dimerization protein 2) double knockout mice (17).

### ATF3-Overexpressing HDFs Inhibit Melanoma Cell Growth and Migration Through a Paracrine Signaling Pathway

**Figure 3:**
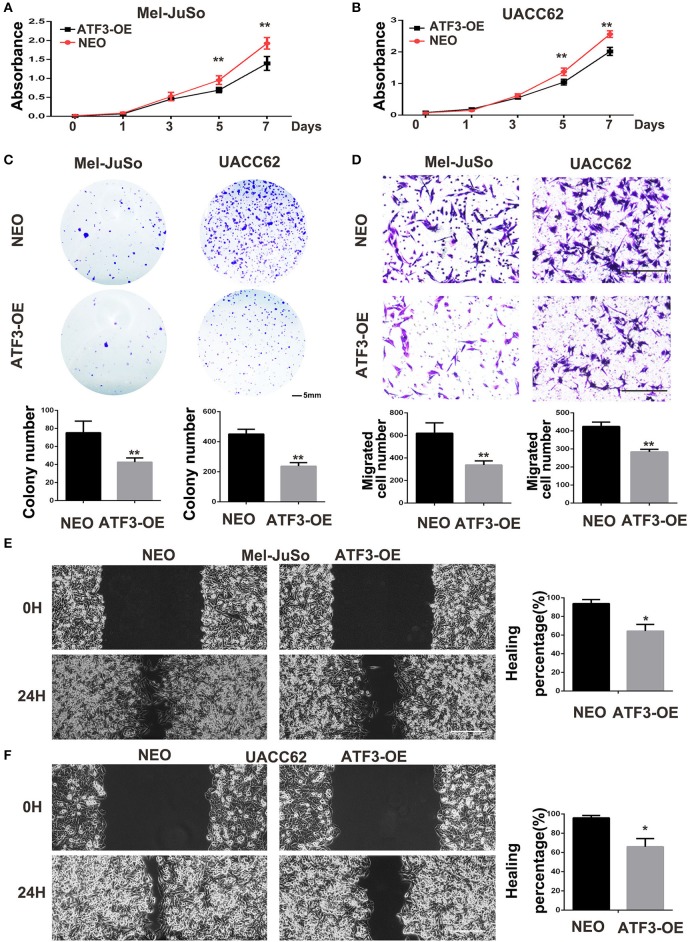
Conditioned medium (CM) derived from ATF3-overexpressing HDFs inhibited melanoma cell growth and migration. CM derived from either ATF3-OE or control NEO HDFs were used for all conditions. **(A,B)** The proliferation of melanoma cells was analyzed at the indicated times. **(C)** Colony formation assay was performed; the lower panel graphs show the average number of colonies formed in each group. **(D)** Melanoma cells that had migrated after 24 h of culture; the average numbers of migrated cells are shown in the lower panel graphs. **(E,F)** Wound healing assay, images of the cells right after scratching (0H) and 24 h after scratching (24H) are shown in the left panel and percentages of wound closure at 24 h are shown in the right panel graphs. Data are presented as the mean ± standard deviation, *p < 0.05, **p < 0.01. The scale bars represent 5 mm **(C)** and 100 μm **(D,E)**.

The above co-culture assay results suggested that the ATF3-overexpressing HDFs inhibited melanoma cell growth and migration through a paracrine signaling pathway. To further support this hypothesis, we grew melanoma cells in conditioned medium (CM) collected from cultures of ATF3-overexpressing HDFs. We found that CM from ATF3-overexpressing HDFs significantly decreased the growth of Mel-JuSo and UACC62 cells compared with CM from NEO-expressing control HDFs (Figures 3A,B). In addition, a cell colony assay showed that CM derived from ATF3-overexpressing HDFs inhibited colonic growth of melanoma cells significantly (Figure 3C). Next, we tested whether CM affects melanoma cell migration. Indeed, CM from ATF3-overexpressing HDFs inhibited migration of both Mel-JuSo and UACC62 melanoma cells compared with CM from NEO-expressing HDFs (Figure 3D), which was further confirmed by a scratch migration assay (Figures 3E,F). Taken together, these data suggest that overexpression of ATF3 in HDFs decreases the secretion of soluble factors that play crucial roles in melanoma cell growth and migration.

### Overexpression of ATF3 Significantly Decreased the Expression of IL-6, IL-8, and Other Cytokines in HDFs

**Figure 4:**
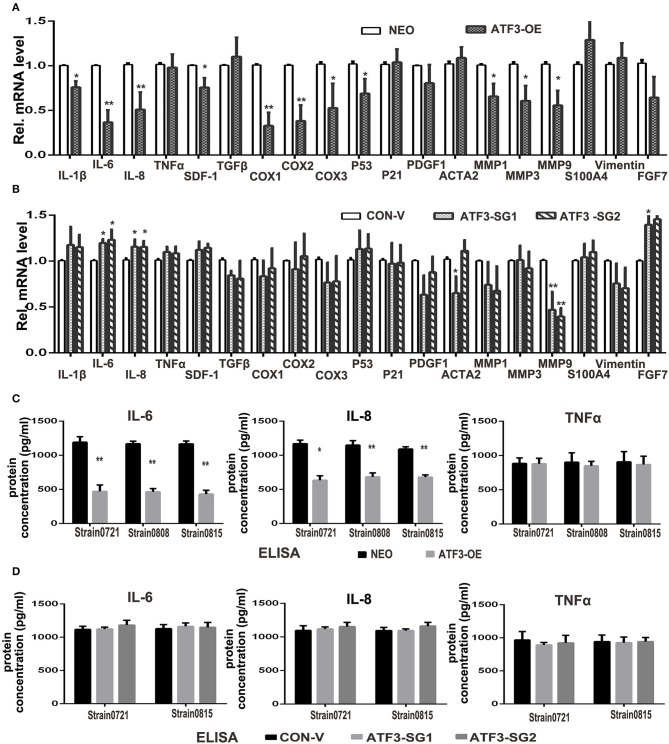
ATF3 overexpression suppressed expression of pro-inflammatory cytokines IL-6 and IL-8 in HDFs. **(A)** The mRNA levels of cytokine, growth factor and CAF-related genes were measured by qRT-PCR in ATF3-OE HDFs and control NEO HDFs. **(B)** The mRNA levels of the same genes as in **(A)** were measured by qRT-PCR in two HDF pools with CRISPR/Cas9-mediated ATF3 deletion by independent small guide RNAs (ATF3-SG1 or ATF3-SG2) and compared with the mRNA levels in control HDFs transduced with empty vector (CON-V). **(C,D)** Protein concentrations of IL-6, IL-8 and TNFα measured by ELISA in CM derived from ATF3-OE or control NEO HDFs **(C)** and in CM from HDFs with CRISPR/Cas9-mediated deletion of ATF3 (ATF3-SG1 or ATF3-SG2) or control vector (CON-V) **(D)**. Data are presented as the mean ± standard deviation, *p < 0.05, **p < 0.01.

ATF3 has previously been shown to downregulate expression of many cytokines including IL-6 and IL-8 (33). Therefore, we examined the effects of ATF3 levels on the expression of potentially relevant cytokines and growth factors in HDFs. First, qRT-PCR analysis showed that the expression levels of interleukin genes such as IL-1β, IL-6 and IL-8, as well as cyclooxygenase genes COX1 and COX2, are significantly lower in ATF3-overexpressing HDFs compared with control HDFs (Figure 4A). To test whether depletion of ATF3 could affect the expression of these genes in HDFs, we performed qRT-PCR analysis of the genes in *ATF3*-KO HDFs. Interestingly, we found that only IL-6 and IL-8 expression increased significantly after ATF3 deletion (Figure 4B), which could also explain why the ATF3 knockout HDFs did not have a significant effect on melanoma cell growth and migration (Supplementary Figure 2). Secondly, enzyme-linked immunosorbent assays (ELISA) were performed to measure cytokine protein levels in CM from HDF cultures. ATF3 overexpression resulted in significantly reduced levels of IL-6 and IL-8, but did not affect TNFα levels in CM, consistent with the qRT-PCR results for those cytokines (Figure 4C). The inhibition of IL-6 levels by ATF3 overexpression was especially notable (3-fold decreased, Figure 4C, left graph). Again, the protein levels of IL-6, IL-8, and TNFα in CM were not affected by ATF3 deletion in HDFs (Figure 4D), differing somewhat from the small but significant increases in IL-6 and IL-8 mRNAs after ATF3 deletion. Taken together, the data above suggest that ATF3 overexpression in HDFs markedly inhibits the expression and secretion of cytokines including IL-6 and IL-8.

### Suppression of IL-6 Expression Is Crucial to the Inhibitory Effects of ATF3-Overexpressing HDFs on Melanoma Cell Growth and Migration

**Figure 5:**
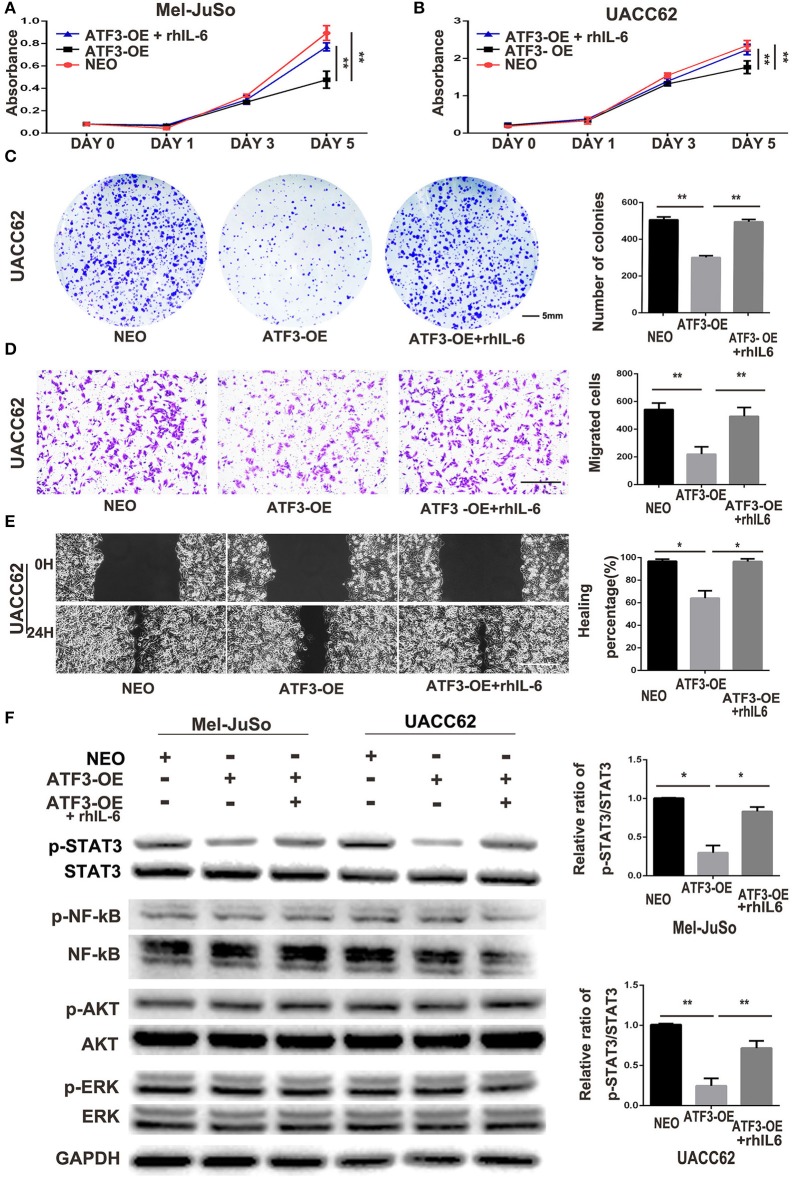
ATF3-overexpressing HDFs inhibit melanoma cell proliferation and migration through regulation of the IL-6/STAT3 pathway. **(A–F)** CM derived from either ATF3-OE or control NEO HDFs, or from ATF3-OE HDFs incubated with 10 ng/ml recombinant human IL-6 (ATF3-OE + rhIL-6) were used to culture melanoma cell lines Mel-JuSo and UACC62 for analysis of growth **(A,B)**, colony formation **(C)**, transwell-migration **(D)**, wound healing **(E)**, and phosphorylated (active) states of the indicated signaling proteins **(F)**. Quantification results for C-F assays are shown in the graphs at right. The relative levels of phosphorylated STAT3 in **F** were normalized to the respective total STAT3 bands. The scale bars in the images represent 5 mm **(C)** and 100 μm **(D,E)**. Data are presented as the mean ± standard deviation, *p < 0.05, **p < 0.01.

Cytokines produced in the tumor microenvironment have been shown to play important roles in different stages of cancer development (3, 34). Thus, it is likely that the decreased production of cytokines in HDFs overexpressing ATF3 may be critical for the inhibition of melanoma cell growth and migration. IL-6, a pro-inflammatory cytokine, has been well-documented to play important roles in development and progression of melanoma (35), and its expression was markedly reduced in ATF3-overexpressing HDFs. Therefore, we tested whether replenishment of IL-6 could counteract the effects of ATF3-overexpressing HDFs on melanoma cell growth and migration. We added recombinant human IL-6 protein (rhIL-6) to CM derived from HDFs overexpressing ATF3 and performed growth, colony formation and migration assays. We found that addition of rhIL-6 to CM from ATF3-overexpressing HDFs reversed the inhibitory effect of the CM on proliferation of melanoma cells (Figures 5A,B) and restored the colony growth of both UACC62 (Figure 5C) and Mel-JuSo (Supplementary Figure 3A) cells to control levels. Finally, both cell migration and wound healing assays showed that addition of rhIL-6 blocked the inhibitory effects of CM from ATF3-overexpressing HDFs on melanoma cell migration (Figures 5D,E, Supplementary Figures 3B,C). Taken together, these data suggest that suppression of IL-6 expression plays a crucial role in the inhibitory effect of ATF3-overexpressing HDFs on melanoma cell proliferation and migration.

It is well-known that IL-6 drives many “hallmarks” of cancer through downstream activation of the Janus kinase/signal transducer and activator of transcription 3 (JAK/STAT3) signaling pathway (36, 37). In agreement with the previous studies, we found that phosphorylation of STAT3 in both Mel-JuSo and UACC62 melanoma cells decreased dramatically after culture with CM derived from ATF3-overexpressing HDFs. Interestingly, other signaling pathways including the NF-κB, PI3K/AKT, and MAPK pathways, were not altered in the melanoma cells cultured with CM from ATF3-overexpressing HDFs (Figure 5F). Taken together, these data suggest that decreased production of IL-6 in ATF3-overexpressing HDFs contributes to inhibition of melanoma cell proliferation and migration by blocking the STAT3 pathway.

### HDFs With High Expression of ATF3 Repress Melanoma Cell Tumor Formation and Growth *in vivo*

**Figure 6:**
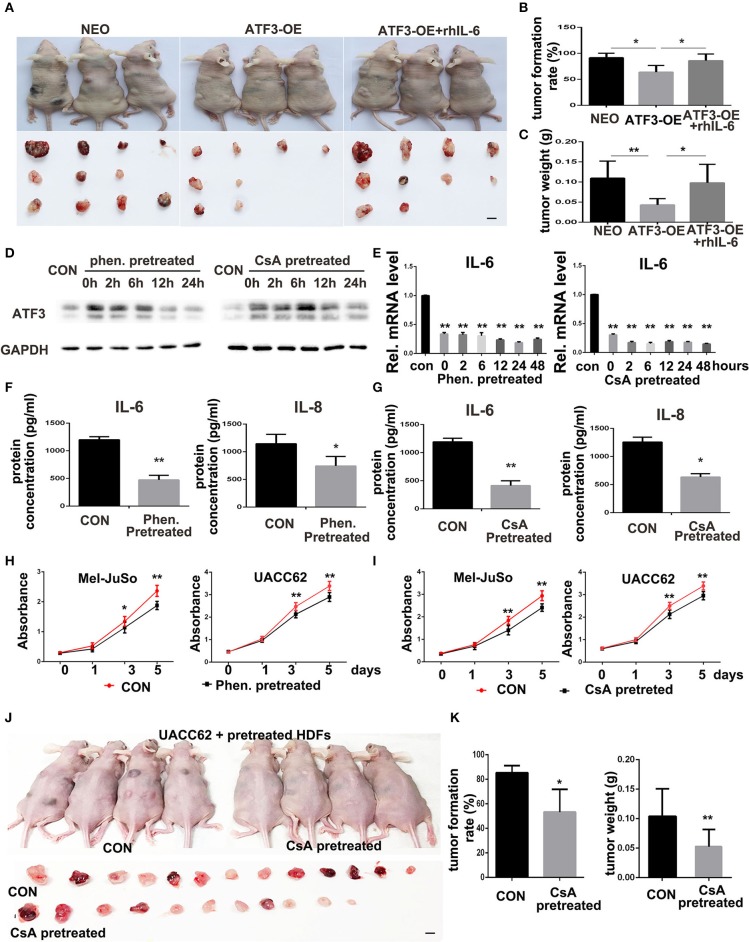
ATF3-overexpressing HDFs inhibit melanoma formation and growth *in vivo*. **(A)** Mice and tumors collected at 3 weeks after grafting with the indicated conditions. rhIL-6 (100 ng/mouse/day) was administrated by intraperitoneal injection. **(B)** The percentage of tumor formation. **(C)** The average weight of tumors. **(D,E)** HDFs pretreated with 1.5 mM phenformin (Phen.), 10 μM CsA, or DMSO vehicle (CON) for 24 h were collected at the indicated times for analysis of ATF3, IL-6, and IL-8. **(F,G)** CM derived from pretreated HDFs was analyzed for IL-6 and IL-8 levels. **(H,I)** CCK-8 assay after culturing melanoma cells with CM from pretreated HDFs at the indicated time points. **(J,K)** Mice and tumors collected 3 weeks after grafting as indicated conditions. The percentage of tumor formation and the average tumor weight are shown in **(K)**. The scale bars: 5 mm. Data are presented as the mean ± standard deviation, *p < 0.05, **p < 0.01.

To illustrate the *in vivo* biological function of HDFs with high level expression of ATF3, UACC62 melanoma cells mixed with ATF3-overexpressing HDFs or NEO-expressing control HDFs were subcutaneously injected into the back skin of nude/nude mice. The mice injected with ATF3-overexpressing HDFs were then divided into two groups with or without intraperitoneal injection of rhIL-6 (Figure 6A). At 3-weeks after injection, the tumors were collected and weighted. The average size of tumors grown with ATF3-overexpressing HDFs (without rhIL-6) was smaller than the average sizes of tumors grown with NEO-expressing control HDFs or with ATF3-overexpressing HDFs plus intraperitoneal injection of rhIL-6 (Figure 6A). The number and average weight of tumors that formed in the mice that received ATF3-overexpressing HDFs without rhIL-6 were significantly lower than in the other two groups (Figures 6B,C). These *in vivo* experiments validated our *in vitro* findings by showing that HDFs with high expression of ATF3 can repress melanoma cell tumor formation and growth, probably through negative regulation of IL-6 production.

### Induction of ATF3 Expression in HDFs Can Inhibit Melanoma Cell Growth Both *in vitro* and *in vivo*

The *in vivo* inhibitory effects of HDFs with high expression of ATF3 on melanoma formation and growth suggests a potential new approach for treatment of melanoma in the clinic, namely inducing tumor stromal cells to increase ATF3 expression. To test that possibility, we screened several compounds that were previously reported to induce ATF3 expression and to play anti-tumor roles, including cyclosporine A (CsA) (23), cisplatin (38), metformin/phenformin (39), and sulfuretin (40). We found that two of those compounds, CsA and phenformin, can significantly induce expression of *ATF3* mRNA in HDFs (Supplementary Figures 4A,C). We chose 1.5 mM phenformin and 10 μM CsA as the optimal concentrations for further study and found that those concentrations induced ATF3 protein expression within 2 h (Supplementary Figures 4B,D); especially 10 μM CsA treatment could result in about 4-fold induction of ATF3 expression, which is similar to the expression level in ATF3 overexpressing HDF strain 0808 (Figure 2A). To investigate whether induction of ATF3 expression in HDFs could inhibit melanoma cell growth, we pretreated HDFs with either CsA or phenformin, then removed the drugs and continued culturing the HDFs with complete growth medium. We observed that ATF3 expression levels in HDFs remained high up to 24 h after removal of the compounds (Figure 6D) and, importantly, both compounds suppressed IL-6 and IL-8 mRNA levels for at least 48 h after their withdrawal (Figure 6E), which was confirmed by ELISA (Figures 6F,G). Then we found that the suppression of IL-6 expression in HDFs pretreated with either CsA or phenformin was abolished by deletion of ATF3 (Supplementary Figure 5), suggesting it depends on the expression of ATF3. Next, to test whether HDFs pre-treated with CsA or phenformin can affect melanoma cell growth, we cultured melanoma cells in CM derived from HDFs that had been treated with either phenformin or CsA for 24 h followed by continuation of the HDF culture in the absence of drug for another 48 h before collection of the CM. We found that CM from HDFs pre-treated with phenformin or CsA can significantly inhibit the growth of both Mel-JuSo and UACC62 melanoma cells (Figures 6H,I). Next, HDFs were pretreated with CsA or DMSO vehicle, mixed with an equal number of UACC62 melanoma cells and injected subcutaneously into the back skin of nude/nude mice. Three weeks after injection, we found that the percentage of mice with detectable tumors, as well as average tumor sizes weights were significantly lower in the mice that received HDFs pretreated with CsA (Figures 6J,K). Taken together, these data suggest that compounds capable of inducing ATF3 expression in HDFs could potentially offer a novel treatment strategy for melanoma *in vivo*.

## Discussion

First described in Paget's “seed and soil” theory (41), tumor stromal cells have been well-recognized to play critical roles in tumor initiation, progression and metastasis (42). Activating transcription factor 3 (ATF3), a eukaryotic stress response gene, is a key transcription factor and a member of the ATF3/cAMP-response element-binding protein family (16, 43). It has been extensively studied in tumor cells, and the role of ATF3 in tumor cells has been shown to be cell type-specific. However, the role of ATF3 in tumor stromal cells has been little explored so far. Just recently, Kim et al. investigated ATF3 function in stromal fibroblasts of skin SCC and found *ATF3* deletion in skin fibroblasts were prone to develop aggressive chemically-induced skin tumors with enhanced CAF activation (33). Avraham et al. reported that double deletion of *ATF3* and *JDP2* in mouse CAFs promoted tumor growth (17). Our present study revealed that ATF3 expression is low in stromal cells of clinical melanoma tissues, and human dermal fibroblasts with overexpression of ATF3 can inhibit melanoma cell growth and migration both in culture and *in vivo* in a mouse model. Our findings further support that ATF3 in stromal fibroblasts plays a tumor suppressing function in the skin. It will be interesting to further investigate in future studies how ATF3 content in stroma cells is involved in different stages of melanomagenesis and progression. We also observed strong nuclear staining of ATF3 in melanoma tumor cells (Figure 1), and we are investigating the role of intratumoral ATF3 in melanoma growth and progression in a separate study.

Stromal cells communicate with tumor cells by either direct cell-cell contact or by soluble factors through paracrine signaling (44). The soluble factors secreted by stromal cells, including cytokines and growth factors, play profound roles in tumor cell growth and migration. Our co-culture and conditioned medium assays demonstrated that ATF3-overexpressing HDFs blocked both Mel-JuSo and UACC62 melanoma cell proliferation and migration through a paracrine signaling pathway. It is well-known that ATF3, a transcriptional repressor, negatively regulates expression of numerous cytokines in immune cells (16, 45, 46); also skin dermal fibroblasts with ATF3 deletion upregulated CAF effector genes including cytokines and matrix metalloproteases (33). Therefore, we examined these genes in our system and confirmed that expression of CAF-related cytokines such as IL-6, IL-8 and COX1/2 were decreased in ATF3-overexpressing HDFs, and increased in ATF3-deleted HDFs. Among the genes we investigated, the negative regulation of IL-6 expression by ATF3 overexpression in HDFs was the most profound. We found that the addition of recombinant human IL-6 protein *in vitro* and *in vivo* could reverse the inhibition of melanoma cell growth and migration by ATF3-overexpressing HDFs. Furthermore, the JAK/STAT3 pathway, the IL-6 downstream target (47, 48), was downregulated in ATF3-overexpressing HDFs. IL-6 has been shown to play a promoting role in the pathogenesis and development of malignancies in many kinds of tumors, including melanoma, and was also shown to be highly expressed by CAFs contacting different tumor types (34, 49–51). Therefore, we could conclude that the inhibitory effect of ATF3-overexpressing HDFs on melanoma cell growth and migration is mainly through the IL-6/STAT3 pathway. However, we cannot discount that the reductions of other cytokines including IL-8, SDF-1 and COX-1/2 in ATF3-overexpressing HDFs could also contribute to the inhibition of melanoma growth and migration.

With the recognition of the crucial roles of tumor microenvironment stromal cells in tumor development, progression and metastasis, significant efforts have been made in the last two decades to develop strategies that target the tumor stroma to improve cancer therapy, however, completely curative approaches are not currently available (42, 44). Melanoma has been recognized as an exceptionally aggressive and drug-resistant skin cancer. Major advances have been made in therapies for several other cancers, but melanoma remains difficult to manage. Melanoma cells are often characterized by an activated extracellular signal–regulated kinase (ERK) pathway induced by BRAF activating mutations (3, 15). BRAF inhibitors for treatment of melanoma are effective transiently, however, most patients develop resistance. Accumulating evidence suggests that the tumor stroma contributes significantly to this resistance. Therefore, targeting tumor stroma might be a potential strategy to prevent BRAF inhibitor resistance (3). Agents targeting FAP-α, CXCR4/CXCL12, TGF-β, and other signaling in tumor stroma have been under preclinical study or clinical trials for various cancers including melanoma (52, 53). The active mechanisms of these therapeutic approaches are either through directly inhibiting fibroblast growth or through targeting signaling pathways involved in stroma-tumor cell interactions (52, 54). Our study shows not only that artificial overexpression of ATF3 in normal HDFs suppresses melanoma cell growth but that, importantly, induced expression of ATF3 in HDFs by pretreatment with CsA or phenformin, associated with decreased expression of cytokines, can also inhibit melanoma cell growth both *in vitro* and *in vivo* (Figure 6). Although we cannot exclude the possibility that other factors induced in HDFs by pretreatment with CsA or phenformin are involved in inhibiting tumor cell growth, our results suggest that increased ATF3 expression in HDFs could potentially block tumor cell growth clinically. Our study provides an opportunity to develop a novel strategy for melanoma treatment, to specifically induce ATF3 expression in tumor stromal cells, which could be combined with other conventional and/or innovative treatments to enhance melanoma therapy in the future.

## Data Availability Statement

The datasets generated for this study are available on request to the corresponding author.

## Ethics Statement

All animal studies were approved by the ethics committee of the Hospital of Stomatology, Shandong University (Protocol No. GR201720, Date: 02-27-2017). All of the animal procedures in this study were carried out in accordance with National Institutes of Health Guidelines for the Care and Use of Laboratory Animals and the principles of the Basel Declaration. Foreskin tissues were obtained from discarded hospital specimens without any personal identity information, and clinical nevus and melanoma tissues were obtained from patients who had provided written informed consent in accordance with the Declaration of Helsinki, at Qilu Hospital, Shandong University. The procedures for obtaining those tissues were approved by the Medical Ethical Committee of the School of Stomatology, Shandong University (Protocol No. 20150401, Date: 12-05-2015).

## Author Contributions

XW, CB, and DF designed the experiments. TZ, JW, LX, HL (4th Author), JM, and HL (6th Author) conducted the experiments. TZ and XW analyzed the data and wrote the manuscript. All authors read and approved the final manuscript.

## Conflict of Interest

DF has a financial interest in Soltego, Inc., a company developing SIK inhibitors for topical skin darkening treatments that might be used for a broad set of human applications. DF's interests were reviewed and are managed by Massachusetts General Hospital and Partners HealthCare in accordance with their conflict of interest policies. The remaining authors declare that the research was conducted in the absence of any commercial or financial relationships that could be construed as a potential conflict of interest.
