# Supplementary material for: Up-Regulation of Activating Transcription Factor 3 in Human Fibroblasts Inhibits Melanoma Cell Growth and Migration Through a Paracrine Pathway
Source: Front Oncol. 2020 Apr 21;10:624. doi: 10.3389/fonc.2020.00624 (PMC7187895; doi:10.3389/fonc.2020.00624)
Supplement: Supplementary file 1 [file Data_Sheet_1.DOCX]

Supplementary Material

## Supplementary Figures


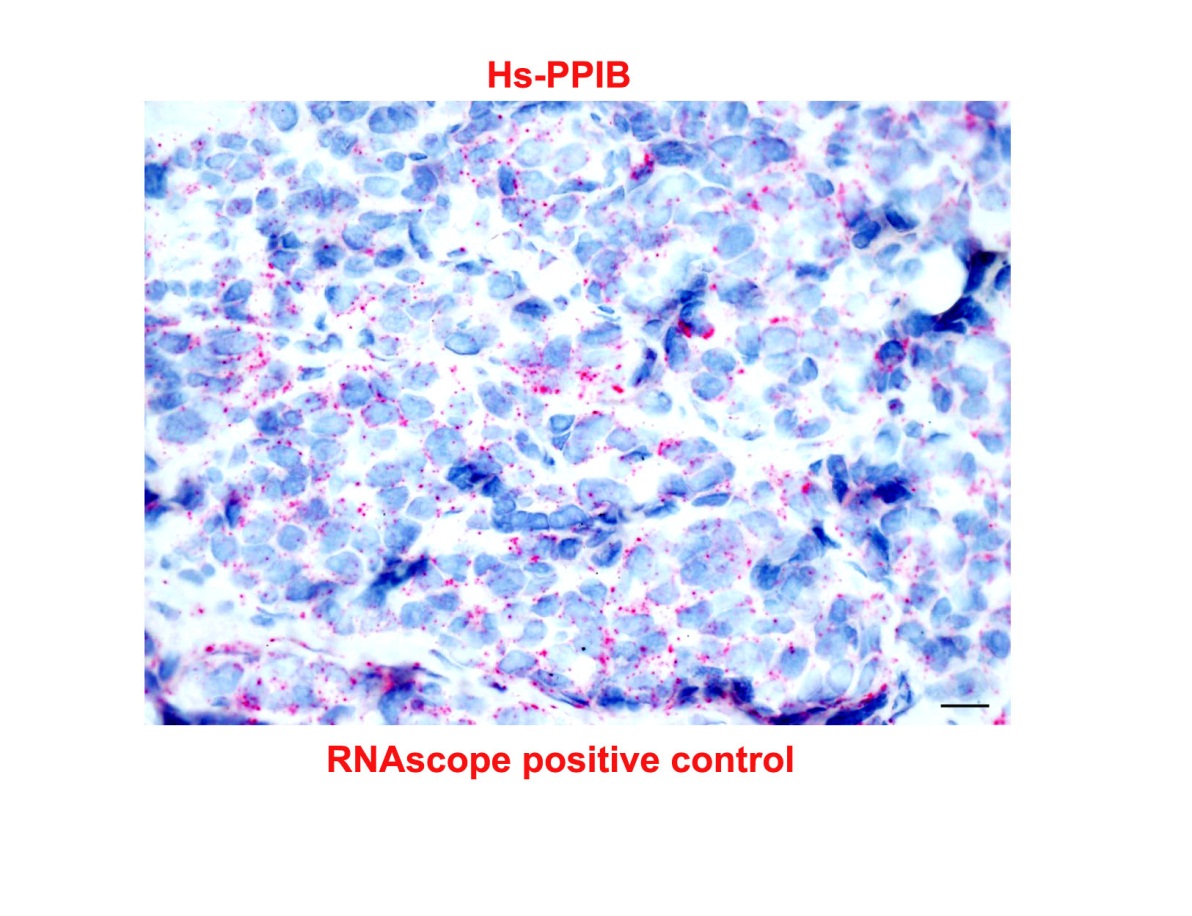


**Supplementary Figure 1. The positive control staining for RNAscope *in situ* hybridization.**

RNAscope Probe Hs-PPIB (Human-Peptidylprolyl Isomerase B) was used as positive control for staining (Red points) melanoma specimen sections. Hematoxylin was used for counterstaining. Bar represents 20 μm.

**
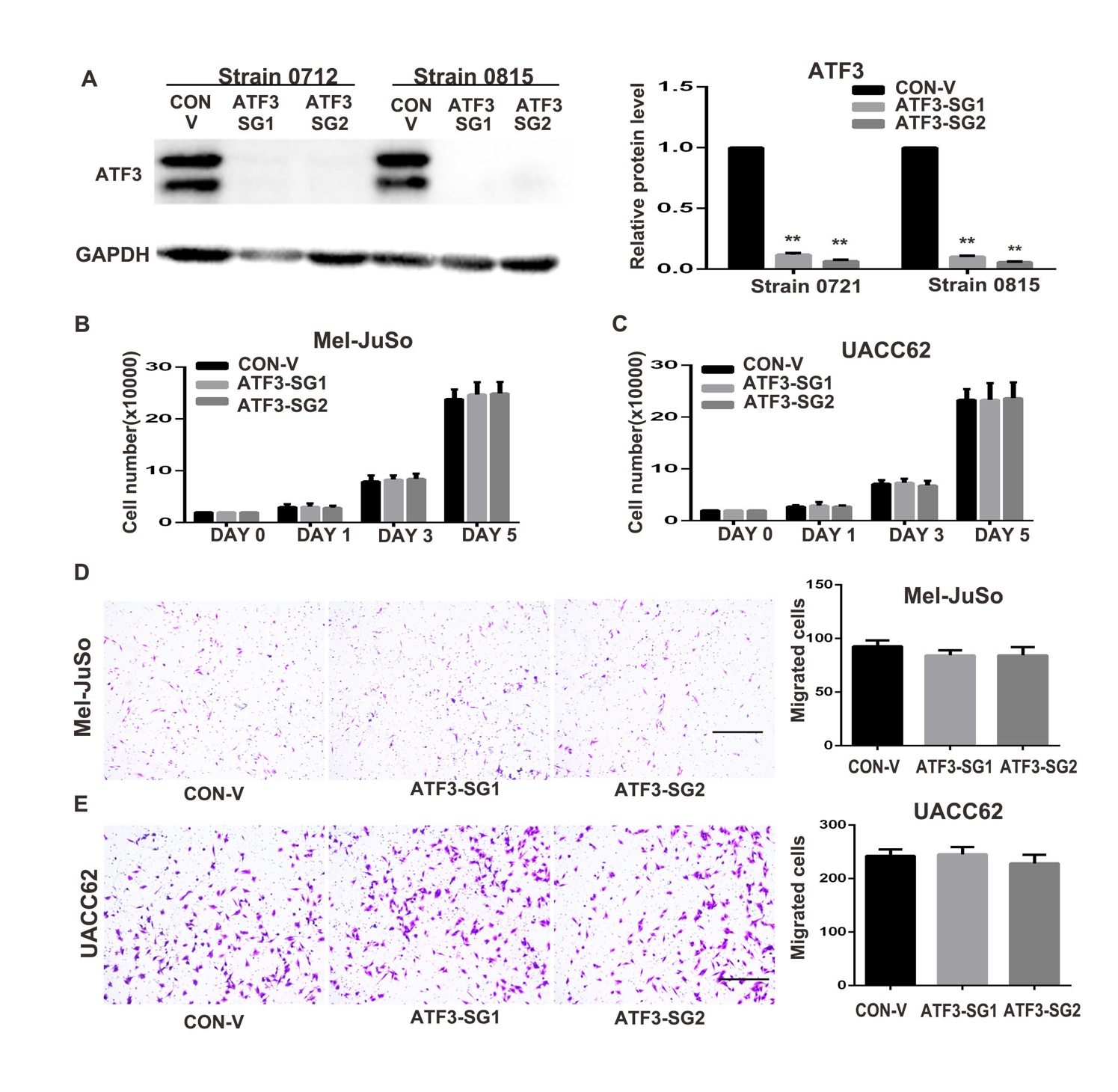
**

**Supplementary Figure 2. HDFs with deletion of ATF3 did not significantly affect melanoma cell growth and migration.**

**A.** ATF3 protein levels were detected by Western blot in 2 HDF strains 48 h after infection with CRISPR/Cas9 lentivirus expressing ATF3-targeting small guide RNA ATF3-SG1 or ATF3-SG2, or with empty vector as control (CON-V). GAPDH was used as loading control. The quantified ATF3 protein levels relative to the levels in CON-V are shown in the right panel graph. **B-C**. The proliferation of Mel-JuSo (B) and UACC62 (C) cells was analyzed by cell counting at the indicated times after co-culture with ATF3-deleted HDFs (ATF3-SG1 or ATF3-SG2) or control HDFs (CON-V). **D-E**. Mel-JuSo (D) and UACC62 (E) cells that had migrated after co-culture with ATF3-deleted HDFs (ATF3-SG1 or ATF3-SG2) or control HDFs (CON-V) for 24 hours (left panels); the numbers of migrated cells in the different groups are shown in the right panel graphs. Data are presented as the mean ± standard deviation, **p<0.01 compared with the corresponding control group. Bars in D and E represent 100 μm.


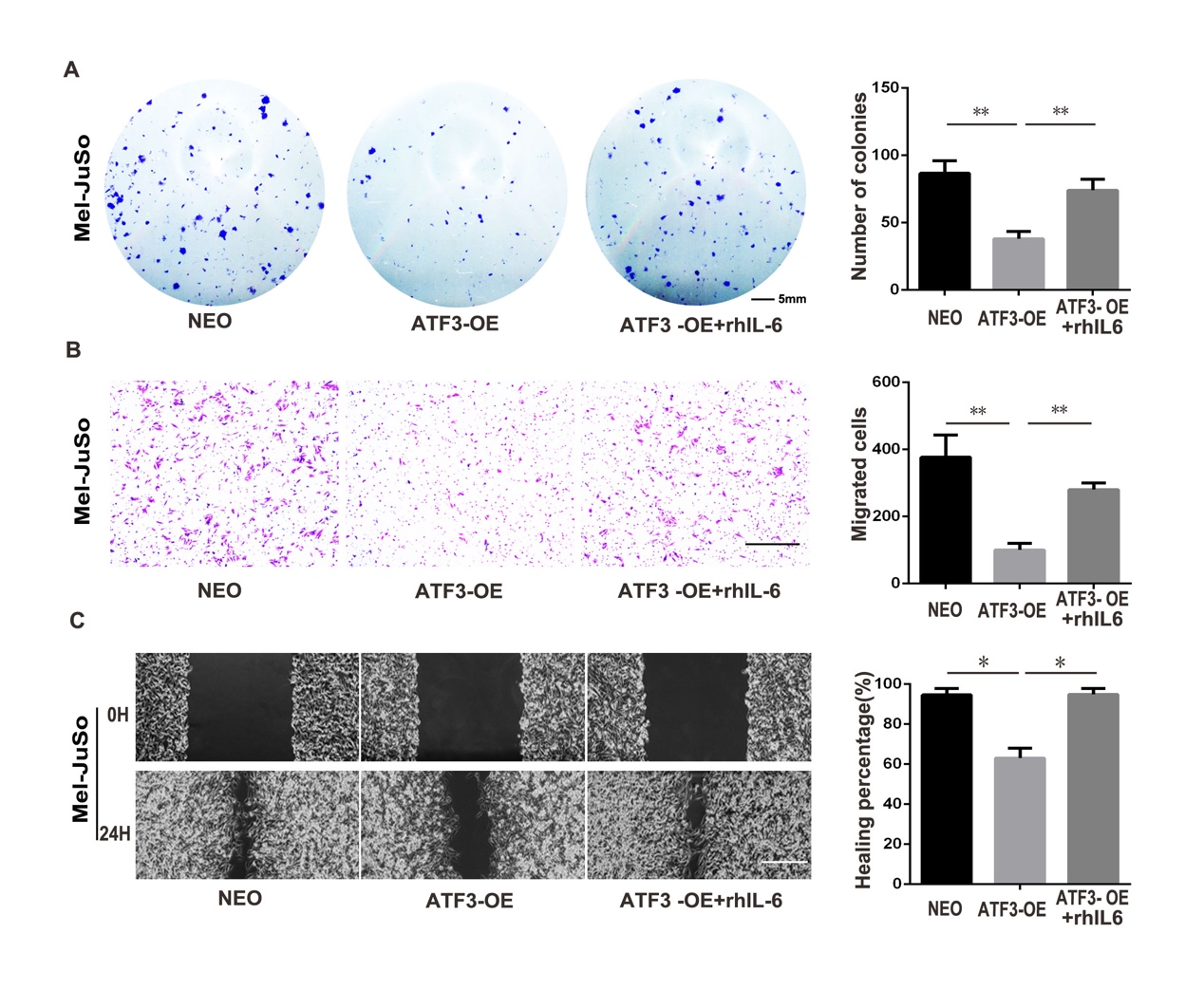


**Supplementary Figure 3. The inhibitory effect of ATF3-overexpressing HDFs on Mel-JuSo cell proliferation and migration is abolished by rhIL-6.**

**A.** The colony formation assay was carried out with Mel-JuSo melanoma cells cultured with the same three HDF CM conditions as in Fig. 5A and B. Images of colonies are shown in the left panels and the average of number of colonies in each condition is shown in the right panel graph. **B.** The same three CM conditions were used in a cell migration assay of Mel-JuSo. Images of the migrated Mel-JuSo cells after 24 h of culture with the indicated CM conditions are shown in the left panels and the average number of migrated cells in each group is shown in the right panel graph. **C.** The same three CM conditions were also used in a wound healing assay of Mel-JuSo cells. Images of cells right after scratching (0H) and 24 h after scratching (24H) are shown in the left panel and the percentage of wound closure in each group at 24 h is shown in the right panel graph. Data are presented as the mean ± standard deviation, **p<0.01 compared with the corresponding control group. The scale bars represent 5 mm (A) and 100 μm (B,C).


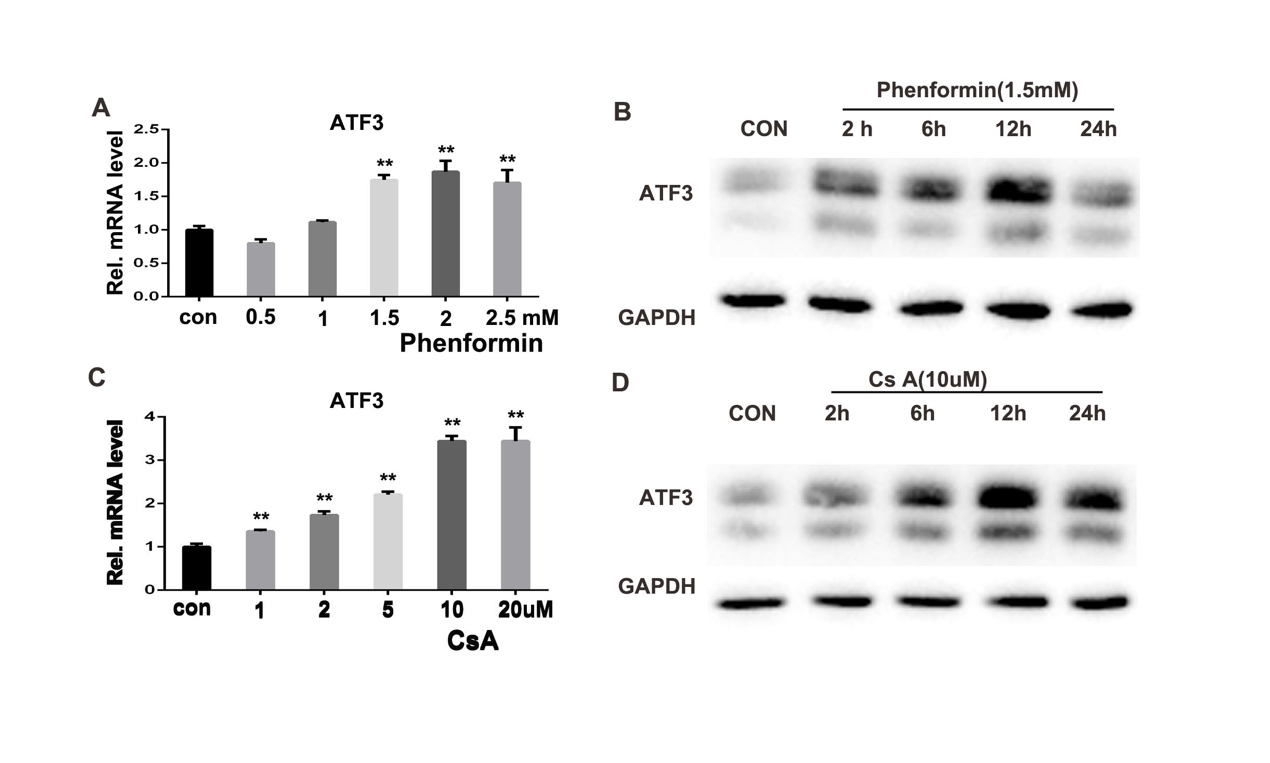


**Supplementary Figure 4. Phenformin and CsA can induce ATF3 expression in HDFs.**

**A.** HDFs were treated with the indicated concentrations of phenformin or DMSO vehicle (con) for 24 h, then the cells were collected for analysis of ATF3 mRNA level by qRT-PCR. The ATF3 mRNA levels normalized to the respective levels of housekeeping mRNA human RPLPO were plotted relative to the DMSO control (con). **B.** HDFs were treated with 1.5 mM phenformin and collected at the indicated time points for analysis of ATF3 protein levels by Western blot. GAPDH was used as loading control. **C.** HDFs were treated with the indicated concentrations of CsA or DMSO vehicle (con) for 24 h, then the cells were collected for analysis of ATF3 mRNA level by qRT-PCR. The ATF3 mRNA levels normalized to the respective levels of housekeeping mRNA human RPLPO were plotted relative to the DMSO control (con). **D.** HDFs were treated with 10 μM CsA and collected at the indicated time points for analysis of ATF3 protein levels by Western blot. GAPDH was used as loading control. Data are presented as the mean ± standard deviation, **p<0.01 compared with the control group.


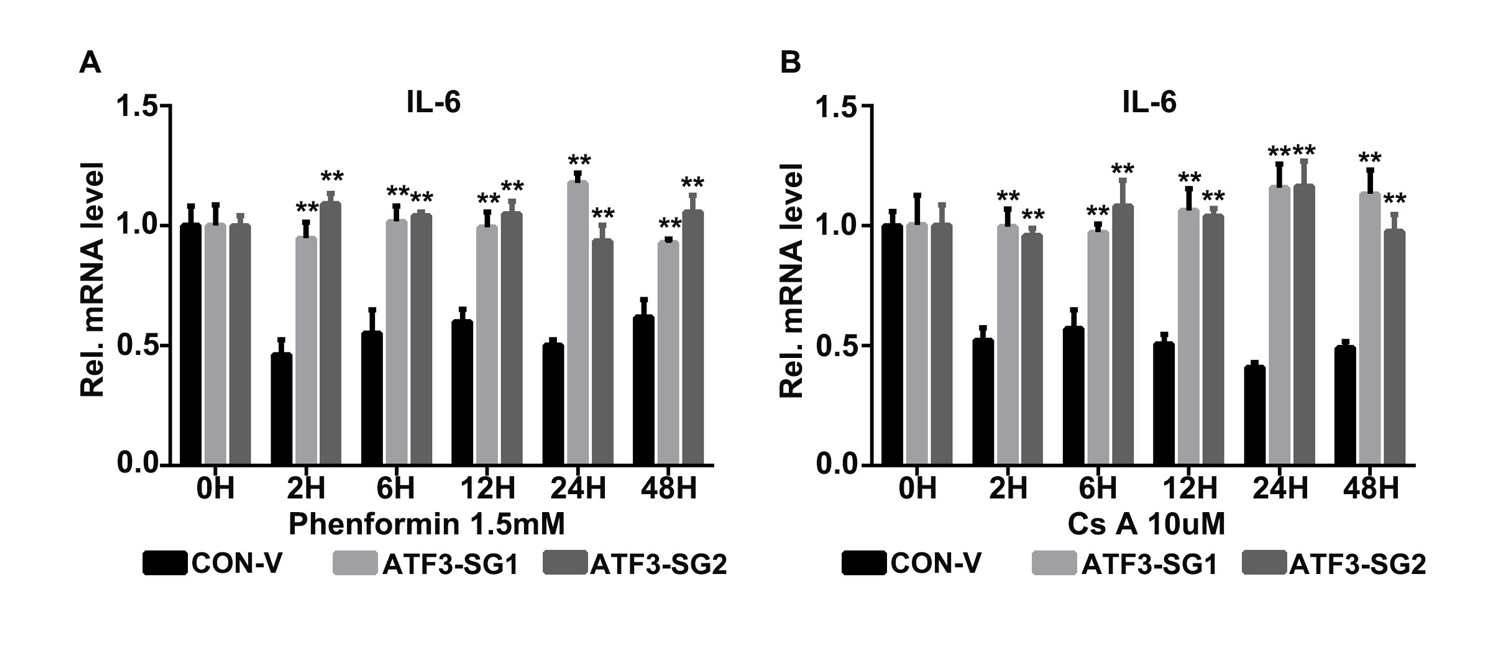


**Supplementary Figure 5. Downregulation of IL-6 expression in HDFs pretreated with CsA or phenformin depends on ATF3 expression**

**A-B**: HDFs infected with CRISPR/Cas9 lentivirus expressing ATF3-targeting small guide RNA ATF3-SG1 or ATF3-SG2, or with empty vector as control (CON-V) were verified for deletion efficiency of ATF3 (Figure S2) and then treated with the indicated concentrations of phenformin (**A**) or CsA (**B**). The cells were collected for analysis of IL-6 mRNA level by qRT-PCR at the indicated times. The IL-6 mRNA levels were normalized to the respective levels of housekeeping gene human RPLPO. Data are presented as the mean ± standard deviation; **p<0.01 when compared to the corresponding control group (CON-V) at each time point.

**Supplementary Tables**

**Supplementary Table 1. oligo sequences of genes for RT-PCR analysis**

| **Gene** | **Forward (5’-3’)** | **Reverse (5’-3’)** |
| --- | --- | --- |
| ATF3 | GTCCATCACAAAAGCCGAGG | GCACTCCGTCTTCTCCTTCT |
| IL-1β | CGCCAGTGAAATGATGGCTT | ATCCAGAGGGCAGAGGTCC |
| IL-6 | TACATCCTCGACGGCATCTC | AGTGCCTCTTTGCTGCTTTC |
| IL-8 | GCCTTCCTGATTTCTGCAGC | TTGGGGTGGAAAGGTTTGGA |
| TNFα | GCTGCACTTTGGAGTGATCG | CTTGTCACTCGGGGTTCGAG |
| SDF-1 | CAGAGCCAACGTCAAGCATC | CCACGTCTTTGCCCTTTCATC |
| TGFβ | CAGCAGGGATAACACACTGC | CATGAGAAGCAGGAAAGGCC |
| COX 1 | CTGGCGCTCAGCCATACAG | CGCACTTATACTGGTCAAATCCC |
| COX 2 | CAGATCATAAGCGAGGGCCA | TGAAAAGGCGCAGTTTACGC |
| COX 3 | ATGCTGACTATGGCTACAAAAGC | TCGGGCAATCATCAGGCAC |
| P 53 | TGGCCATCTACAAGCAGTCA | ATAAGATGCTGAGGAGGGGC |
| P 21 | AGTCAGTTCCTTGTGGAGCC | GCATGGGTTCTGACGGACAT |
| PDGF 1 | CTGAGGTTCAGAGCGCGG | CACCTCCACCAAGTCCTCG |
| ACTA2 | ATCCCCGGGACTAAGACGG | AGAGCCATTGTCACACACCA |
| MMP 1 | CCAGGTATTGGAGGGGATGC | GTCCAAGAGAATGGCCGAGT |
| MMP 3 | ACTCACATTCTCCAGGCTGT | TGGGCCAAAACATTTCCAGG |
| MMP 9 | TCTTCCCTGGAGACCTGAGA | TTTCGACTCTCCACGCATCT |
| S100A4 | GTACTCGGGCAAAGAGGGTG | TTGTCCCTGTTGCTGTCCAA |
| Vimentin | AATGGCTCGTCACCTTCGTG | CAGAGAAATCCTGCTCTCCTCG |
| FGF 7 | TGTCGAACACAGTGGTACCTG | TTGCCACAATTCCAACTGCC |
| H36B4 | GCAATGTTGCCAGTGTCTGT | GCCTTGACCTTTTCAGCAAG |
| ATF3-sgRNA1 | CACCGTGAGCCCGGACAATACACGT | CACTCGGGCCTGTTATGTGCACAAA |
| ATF3-sgRNA2 | CACCGTCAAACACCAGTGACCCAGG | CAGTTTGTGGTCACTGGGTCCCAAA |

**Supplementary Table 2. Antibody information**

**Primary Antibody**

| Protein | Manufacturer | Cat. No. | Dilution |
| --- | --- | --- | --- |
| Anti-ATF3 Antibody | Abcam | 207434 | 1:2000 |
| Anti-ATF3 Antibody | Abcam | 216569 | 1:300 |
| Vimentin Mouse mAb | CST | 3390 | 1:500 |
| p44/42 MAPK (Erk1/2) Rabbit mAb | CST | 4695 | 1:1000 |
| Phospho-p44/42 MAPK (Erk1/2)Rabbit mAb | CST | 4370 | 1:2000 |
| Akt (pan) Rabbit mAb | CST | 4691 | 1:1000 |
| Phospho-Akt Rabbit mAb | CST | 4060 | 1:2000 |
| Stat3 Rabbit mAb | CST | 12640 | 1:1000 |
| Phospho-Stat3 Rabbit mAb | CST | 94994 | 1:1000 |
| NF-κB p65 Rabbit mAb | CST | 8242 | 1:1000 |
| Phospho-NF-κB p65 Rabbit mAb | CST | 3033 | 1:1000 |
| S-100 Rabbit polyclonal antibody | ZSGB-BIO | ZA-0225 | 1:200 |
| GAPDH | Biolegend | 649203 | 1:5000 |

**Secondary Antibodies**

| DyLight488 goat anti-mouse IgG(H+L) | Multi Science | GAM4882 | 1:500 |
| --- | --- | --- | --- |
| DyLight594 goat anti-rabbit IgG(H+L) | Multi Science | GAR5942 | 1:500 |
| [HRP-conjugated Affinipure Goat Anti-Rabbit IgG(H+L)](http://www.ptgcn.com/products/HRP-conjugated-Affinipure-Goat-Anti-Rabbit-IgG-H-L--secondary-antibody.htm) | Proteintech | [SA00001-2](http://www.ptgcn.com/Products/HRP-conjugated-Affinipure-Goat-Anti-Rabbit-IgG-H-L--secondary-antibody.htm) | 1:5000 |
| [HRP-conjugated Affinipure Goat Anti-Mouse IgG(H+L)](http://www.ptgcn.com/products/HRP-conjugated-Affinipure-Goat-Anti-Mouse-IgG-H-L--secondary-antibody.htm) | Proteintech | [SA00001-1](http://www.ptgcn.com/Products/HRP-conjugated-Affinipure-Goat-Anti-Mouse-IgG-H-L--secondary-antibody.htm) | 1:5000 |
